# Vaccine Production to Protect Animals Against Pathogenic Clostridia

**DOI:** 10.3390/toxins11090525

**Published:** 2019-09-11

**Authors:** Nicolas E. Zaragoza, Camila A. Orellana, Glenn A. Moonen, George Moutafis, Esteban Marcellin

**Affiliations:** 1 Australian Institute for Bioengineering and Nanotechnology (AIBN), The University of Queensland, Brisbane, QLD 4072, Australia; n.zaragoza@uq.edu.au (N.E.Z.); c.orellana@uq.edu.au (C.A.O.); 2 Zoetis, 45 Poplar Road, Parkville VIC 3052, Australia; glen.moonen@zoetis.com (G.A.M.); George.moutafis@zoetis.com (G.M.)

**Keywords:** *Clostridium*, clostridia diseases, vaccine production, toxoids, fermentation

## Abstract

*Clostridium* is a broad genus of anaerobic, spore-forming, rod-shaped, Gram-positive bacteria that can be found in different environments all around the world. The genus includes human and animal pathogens that produce potent exotoxins that cause rapid and potentially fatal diseases responsible for countless human casualties and billion-dollar annual loss to the agricultural sector. Diseases include botulism, tetanus, enterotoxemia, gas gangrene, necrotic enteritis, pseudomembranous colitis, blackleg, and black disease, which are caused by pathogenic *Clostridium*. Due to their ability to sporulate, they cannot be eradicated from the environment. As such, immunization with toxoid or bacterin-toxoid vaccines is the only protective method against infection. Toxins recovered from *Clostridium* cultures are inactivated to form toxoids, which are then formulated into multivalent vaccines. This review discusses the toxins, diseases, and toxoid production processes of the most common pathogenic *Clostridium* species, including *Clostridium botulinum*, *Clostridium tetani*, *Clostridium perfringens*, *Clostridium chauvoei*, *Clostridium septicum*, *Clostridium novyi* and *Clostridium hemolyticum.*

## 1. Introduction

The genus *Clostridium* comprises an abundant range of anaerobic, spore-forming, rod-shaped, Gram-positive bacteria that can be found in many different environments, such as soil, marine sediments, sewage, decomposed and rusted products, as well as human and animal gastrointestinal tracts and feces [1]. Some *Clostridium* species are of special importance for their ability to infect humans and animals. Infections can cause severe illnesses, generally mediated by the release of potent toxins. Due to its pervasiveness, *Clostridium* infections can occur in multiple ways, including contaminated food containing either vegetative cells, spores, or pre-formed toxin(s) [2]. Moreover, deep wounds, lacerations, or burns with a favorable anaerobic environment are also considered portals of entry for pathogenic *Clostridium* [3].

Some pathogenic *Clostridium* species can reside inside the host as part of the normal microbiome or as latent spores without apparent adverse effects [4,5,6]. However, alterations such as the disruption of the gut microbiota by the use of antibiotics, damage caused by medically induced abortions, parasites, radiotherapy, chemotherapy, or diseases such as cancer or neutropenia can favor *Clostridium* pathogenesis [3]. *Clostridium* exotoxins cause mild to fatal damage, affecting the gastrointestinal tract (enterotoxins), soft-tissues, and organs (tissue-destructive toxins), or causing neuronal dysfunctions (neurotoxins) [7]. *Clostridium botulinum* and *Clostridium tetani* produce two of the most powerful toxins known to man, botulinum and tetanus neurotoxins (BoNT and TeNT), causing botulism and tetanus, respectively [8]. The wide range of toxins produced by different *Clostridium perfringens* toxinotypes are responsible for different diseases, such as mild-food poisoning, enterotoxemia, gas gangrene, and necrotic enteritis [9]. Pseudomembranous colitis, blackleg, black disease, and non-traumatic gas gangrene are also other well-known diseases associated with the toxins produced by other *Clostridium*, such as *Clostridium difficile*, *Clostridium septicum*, *Clostridium chauvoei* and *Clostridium novyi* [10,11,12,13].

Many of these diseases are important human health concerns, such as tetanus in non-developed countries [14], *C. perfringens* foodborne infections [15], or the rising incidence of *C. difficle* (arguably a distinct genus) infections [16]. Infections also hurt the agricultural economic sector. Animal clostridiosis infections are probably more frequent and certainly less reported than human clostridia infections. Practices without the proper hygiene control, such as animal castration, vaccination, foot-trimming, marking, dehorning, dog bites, or the improper care of birth associated wounds are important contributing factors to tetanus, botulism, or gas gangrene in animals [4,17,18,19]. The sudden change in feeding or the improper supplementation of protein and phosphorus can lead to pulpy kidney or botulism [12,18]. Finally, parasites such as liver flukes and coccidia can induce the development of black disease and necrotic enteritis [20,21].

**Figure 1:**
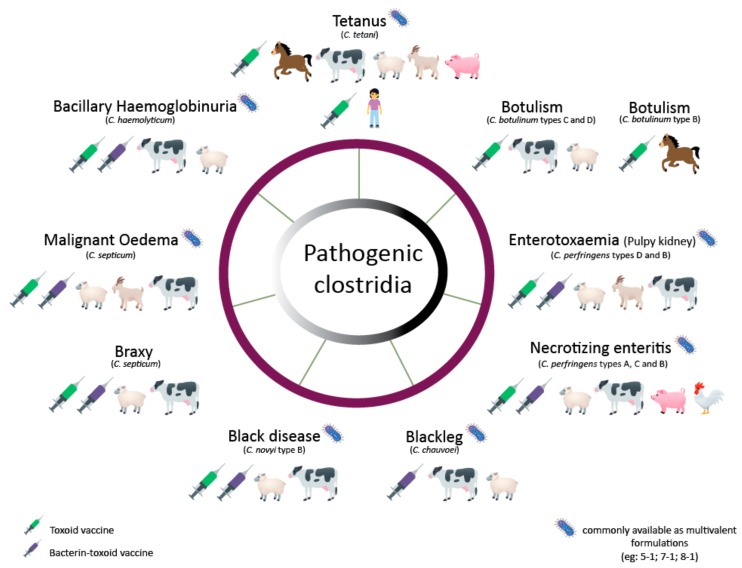
Clostridial diseases and their commercially available vaccines for animals and humans.

Today only tetanus is prevented with a Clostridial vaccine in humans. However, because mortality rates in unvaccinated animals are usually high, there is a large dependence on vaccination in the livestock sector. These vaccines are usually multivalent vaccines combining bacterin-toxoids produced by different *Clostridium* species [22]. Here, we review the production processes of several bacterin-toxoid vaccines to protect against animal pathogenic *Clostridium* species, including *C. botulinum*, *C. tetani*, *C. perfringens*, *C. chauvoei*, *C. septicum*, *C. novyi* and *Clostridium hemolyticum* (Figure 1). Furthermore, a brief description of the diseases caused by the toxins of the latter *Clostridium* and their virulence regulation is also included. The present work does not include *C. difficile*, as there are no commercially available vaccines for the prevention of its disease in animals.

## 2. Clostridium botulinum

Botulism is a deadly neuroparalytic disease caused by the botulinum neurotoxin (BoNT). *C. botulinum* is a species of four different groups of bacteria that share the ability to produce BoNT but differ in metabolic, genetic, and physiological aspects [23,24]. Group I is formed by mesophilic and proteolytic strains that readily digest casein or meat proteins and some carbohydrates, while Group II is psychrotrophic and non-proteolytic but able to ferment different sugars. These two groups account for most of the human botulism cases [25,26]. Group III is proteolytic and saccharolytic and affects animals predominantly [27]. Formerly group IV, now *C. argentinense*, is proteolytic and non-saccharolytic, and has not been associated with botulism cases [28]. The genes encoding BoNTs can be found in the chromosome, plasmids, or phages and are classically classified into seven different serotypes (A–G) [26]. Group I produces serotypes A, B, E, and F, and Group II produces type B, E, and F. Group III produces toxynotypes C and D and *C. argentinense* produces type G (Table 1). More recently, and due to faster and cheaper high-throughput sequencing techniques and bioinformatics, novel BoNTs have been detected, such as BoNT-H and BoNT-X [29,30,31].

*C. botulinum* spores and pre-formed toxins can enter the host in various ways. Human botulism can be acquired due to the ingestion of inadequately cooked, preserved, or refrigerated foods that contain spores or pre-formed toxin. While food-borne botulism is caused by the consumption of contaminated food with pre-formed toxin, infant and adult intestinal botulism are caused by the colonization of the intestinal lumen with *C. botulinum* and consequently in situ production of BoNT [32]. Infant and food-borne botulism are considered the most frequent form of botulism in humans [28,33]. Although less common, botulism can also be contracted through the colonization of wounds, inhalation of the toxin, or by the improper clinical or cosmetic use of the toxin (iatrogenic) [28]. In animals, contaminated or spoiled feed, cannibalism (poultry), bone chewing, and stagnant waters are all sources of infection [18,34]. In Australia, bone chewing in cattle is linked with the lack of phosphorous in the soil of pastoral areas. The use of poultry litter as fertilizer of pastures has also been identified as a major source of botulism outbreaks [35].

BoNT is synthetized as a prototoxin of ~150 kDa that undergoes proteolytic activation resulting in a heavy (~ 100 kDa) and a light chain (~ 50 kDa) connected by a disulphide bond [36]. When BoNT reaches the neuromuscular junction the heavy chain binds to the cholinergic nerve terminal, entering the cells by receptor-mediated endocytosis. Once inside the cell, the metalloprotease activity of the light chain will cleave cytosolic SNARE proteins, preventing the release of neurotransmitter and causing flaccid muscle paralysis. For a more detailed explanation of the mechanisms of action of BoNT, please refer to previous work [37].

At a molecular level, BoNT synthesis is controlled through different regulatory networks. Toxin expression is regulated by the alternative sigma factor BotR, which also regulates the expression of the non-toxic accessory proteins [38]. Two *agr*-like quorum sensing systems have been identified in the proteolytic group of *C. botulinum*—while one controls sporulation, the other one controls toxin production [39]. Two-component systems and the transition state regulator CodY have also been shown to be involved in toxin production regulation [40,41].

### C. botulinum Vaccine Production

In addition to being a major food safety concern, BoNT is also considered a potential bioweapon listed in class A of bioterrorism agents and diseases by the Centers for Disease Control and Prevention (CDC) [42]. Until recently, people at high risk or exposed to the toxin, i.e., researchers and sectors of the military force, were immunized against the disease using the investigational pentavalent botulinum toxoid (PBT) vaccine (BoNT/A–E). However, this vaccine was discontinued as it was losing potency [43]. Human botulism cases, especially those associated with food-borne botulism, have been decreasing since 1950 [44,45], most likely due to the public awareness and good practices in food preparation and sterilization advances in the food industry. BoNT has also been used as a therapeutic agent for treating neuro-ophthalmologic diseases and as a cosmetic treatment for facial rejuvenation (i.e., Botox^®^, Dysport^®^) [46].

Animal botulism outbreaks are more common than in humans and can cause important productivity and economic losses [47,48]. In Europe, animal botulism is considered an emerging disease [49,50]. Contrary to humans, animal vaccines are available in the market from a number of different producers. Livestock can be protected with the bivalent (BoNT/C–D) toxoid vaccine [51,52] and immunization with BoNT/B toxoid vaccine is also recommended for horses [53]. The use of vaccination as post-exposure prophylaxis (PEP) enhanced survival, delayed the progression of characteristic signs, and reduced the severity of the disease in monkeys [54]. In cattle, the use of vaccination is a therapeutic treatment during outbreaks [53].

**Figure 2:**
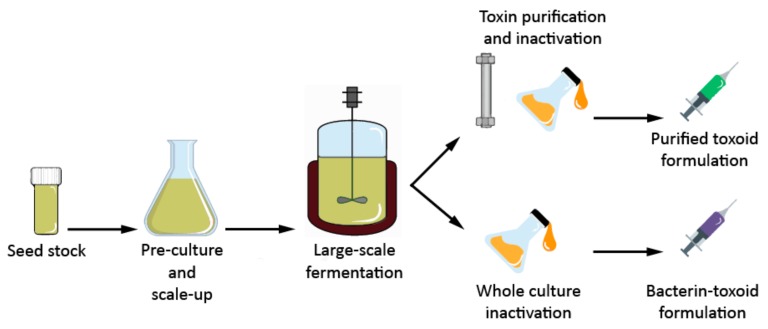
The production steps in a typical clostridial vaccine production process.

BoNT production for the manufacture of toxoid vaccines and for cosmetic and medical use, is achieved by growing *C. botulinum* in fermenters using complex media. Culture medium consists of animal-derived or vegetable peptones, yeast extract, and glucose [55,56]. Maximum toxin concentration can be attained after 24 h of fermentation [57,58]. The inactivation of BoNT for toxoid vaccine production is accomplished by formaldehyde treatment [59] (Figure 2).

Nutritional studies in *C. botulinum* showed that maltose, fructose, mannose, and sucrose are fermented by Group II, while only some strains in Group I ferment maltose and fructose [26]. Glucose is metabolized by both groups and is needed to promote toxin production in Group I [60]. Amino acids, such as arginine and tryptophan, are required by Group I and II, respectively, for growth and toxin synthesis. However, the oversupply of these amino acids has a negative impact on BoNT production [60]. Chemically defined media have been investigated with low success. While showing good growth and sporulation, the amount of toxin produced is considerably reduced [61,62]. Importantly, different fermentation approaches, such as the use of dialysis sacs, have been shown to improve BoNT titers [61].

In recent years, research has focused on recombinant botulinum vaccines that offer an alternative approach to the chemically inactivated toxins. The use of the non-toxic carboxy terminal region of the BoNT heavy chain (HC) for DNA and recombinant protein-based BoNT vaccines has been the base for the design of the new generation of vaccines. Recombinant vaccines expressing the HC fragment of BoNT have also shown to be a potential alternative to toxoid vaccines. Horses vaccinated against recombinant HC BoNT/C were stimulated in the production of antibodies with few adverse reactions compared to the traditional toxoid vaccine [49]. Moreover, a multivalent vaccine comprised of HC-BoNT/A, HC- BoNT/B, and HC-BoNT/E protected mice from a multitoxin challenge [63]. On the other hand, the human recombinant botulinum vaccine A/B (rBV A/B) expressed in the yeast *Pichia pastoris* has been shown to protect mice and non-human primates. However, it was withdrawn from clinical trial phase 3 by the sponsor due to product manufacturing issues and redesign of the study (please refer to: ClinicalTrials.gov, identifier: NCT01940315). For a more detailed review about the new generation of vaccines against botulism, refer to previous work [64].

## 3. Clostridium tetani

*Clostridium tetani* is the etiological agent of tetanus, a fatal neuroparalytic disease caused by the tetanus neurotoxin (TeNT) or tetanospasmin. It affects both humans and animals [65], with approximately 1 million human tetanus cases resulting in 200,000 deaths per year worldwide [66]. The clinical features of the disease are present in records that date back to the 5th century BC [67], yet it remains an important disease in the present day. Presently, the diseases occurs principally in non-industrialized countries that lack vaccination programs and have poor health infrastructure [68]. *C. tetani* is a ubiquitous organism that can be found in the environment irrespective of the geographical location [13,69], and in the gastrointestinal tracts and feces of humans and animals [1]. Tetanus occurs when *C. tetani* spores enter the host through puncture wounds, lacerations, burns, or compound fractures [70,71]. Sadly, the improper sterilization of surgical instruments used to cut the umbilical cord is the principal cause of neonatal tetanus, a relatively common condition in developing countries with poor health infrastructure [14]. In animals, wounds caused by castration, tailing, tagging, shear cuts, and fight bites are associated with *C. tetani* infections [4,72,73,74]. Horses are the most susceptible animals, followed by small ruminants [4,75].

After infection, TeNT is secreted by vegetative cells. TeNT is a zinc metalloprotease that is synthetized as a 150 kDa, single-chain, inactive polypeptide, also called proto-toxin or pTeNT [76]. Like the botulinum toxin, after release, pTeNT undergoes a catalytic process that cleaves the protein into a heavy (~100 kDa) and a light chain (~50 kDa) attached by a disulphide bridge [77]. The heavy chain binds to the motor neuron nerve terminals. Once internalized, it is retrogradely transported to the spinal cord, where it migrates to the inhibitory interneurons [78]. The light chain, containing the zinc metalloprotease activity, cleaves the synaptic vesicle protein synaptobrevin, consequently blocking the release of neurotransmitters [76,77,79,80,81]. The absence of inhibitory impulses leads to continuous muscle contractions primarily seen in the jaw and neck muscles (lockjaw). TeNT not only causes muscle spasms but also affects the neurocirculatory, neuroendocrine, and vegetative nervous systems [65]. The incubation time, which goes from the time of injury to the first symptoms, is between 7 to 10 days [1,67], and it has been reported that shorter incubation periods are associated with higher mortality rates [82].

The tetanus toxin is coded in a plasmid along with the only known toxin regulator, BotR P-21 (formerly TetR) [83]. This regulator belongs to the same subgroup of σ-70 factors of RNA polymerase as BotR (*C. botulinum*), TcdR (*C. difficile*), UviA (*C. perfringens*), and TcsR (*C. sordellii*) [38,84,85]. The collagenase ColT and the cholesterol-dependent cytolysin tetanolysin O are other virulence factors believed to help during infection, contributing to the loss of tissue integrity of the host [13,86]. However, apart from the toxin regulator, studies about the molecular aspects that control *C. tetani* toxinogenesis are non-existent.

### C. tetani Vaccine Production

The mortality rate of tetanus cases in both humans and animals is high [4,87,88]. Nevertheless, tetanus can be effectively prevented by vaccination. The tetanus toxoid vaccine was developed by Ramon and collaborators in the early 1920s [89]. Since the availability of the vaccine, tetanus cases have significantly declined. For example, World War II saw substantially less tetanus-related deaths compared to World War I, when the vaccine was not yet accessible [90]. The incidence of tetanus in animals has also decreased due to consistent immunization programs [88].

The industrial manufacture of tetanus vaccine consists of growing *C. tetani* in batch fermentation for ~160 h [91]. Although *C. tetani* grows for ~48 h, the proteolytic activation of TeNT occurs after the autolysis stage [91]. It is believed that TeNT is released to the extracellular environment by autolysis, as no toxin transporter has been described yet [86]. TeNT is inactivated by formaldehyde to produce the toxoid [89]. Subsequently, the toxoid is further purified from the detoxified cultures by methods such as ammonium sulphate precipitation [92], ultrafiltration [93], tangential flow filtration [94,95], or chromatography [96].

Few variations have been made to the toxin-producing medium designed by Mueller and Miller [97], with the subsequent modifications made by Latham et al. [98]. The complex growth medium contains glucose, vitamins, inorganic salts, and pancreatic digest of casein as the main source of amino acids and peptides [91]. The concentrations of free amino acids in the medium play important roles in toxin production. High concentrations (10 g/L) of free amino acids inhibit toxin production, with glutamate, aspartate, glutamine, asparagine, histidine, and serine having the greatest inhibitory effect [99,100]. Additionally, peptides are also crucial for toxin production, especially histidine containing peptides and short hydrophobic peptides containing proline [101,102]. The pancreatic digest of casein can be fully replaced by soy peptone, avoiding the use of animal-derived products [103,104]. Glucose is another component that has an impact on toxin production, yet it is not fully consumed from the fermentation medium [91]. Latham et al. showed that if the initial concentration of glucose was reduced, toxin was not produced [98]. During the sterilization of the medium, glucose reacts with the amino acids and peptides, forming Maillard reaction products favorable for toxin production [98,105]. Moreover, other fermentation settings, such as fed-batch with the addition of glucose at fixed times [106] or continuous cultures with total consumption of glucose [107], increase toxin production. Zacharias and Björklund also showed the addition of potassium chloride to continuous cultures at pH 7.4 and temperature of 34 °C increased toxin titers [107]. Finally, high concentration of toxin has been obtained by culturing *C. tetani* inside cellophane dialysis sacs [108,109]. However, they could not be implemented at large scale [107].

Oral, nasal, and subcutaneous immunization of cattle with attenuated *Salmonella typhimurium* expressing the C-fragment of TeNT did not elicit full immunity [110]. However, more recently, efforts have focused on developing recombinant vaccines using the tetanus toxin C-fragment of the heavy chain (TeNT-Hc), which has immunogenic properties comparable to the native toxin [111,112,113,114,115]. This subunit vaccine showed good results in mice and rats but required more immunizing doses in monkeys [113]. Although preclinical studies look promising, there is no recombinant tetanus vaccine available in the market yet.

## 4. Clostridium perfringens

*C. perfringens* can produce an arsenal of toxins and virulence factors that participate in the course of infection and pathogenesis. However, no individual strain can produce the entire collection of toxins [9]. Previously classified into five toxinotypes, *C. perfringens* has been reclassified into seven toxinotypes (A–G) depending on the toxin they produce: alpha (CPA), beta (CPB), epsilon (ETX), iota (ITX), *C. perfringens* enterotoxin (CPE), or the necrotic enteritis beta-like toxin (NetB) [9] (Table 1). The majority of *C. perfringens* toxins are encoded in large plasmids ranging from 45 kb to 140 kb, with the exception of CPA and perfringolysin O (PFO), which are located on the chromosome. CPE can also be found in the chromosome or on plasmids. Most of these plasmids are conjugative and have a region known as the tcp locus, which is believed to contribute to the spread of toxin genes and resistance determinants among strains [116]. Most diseases caused by *C. perfringens* isolates are mediated by one or more of these toxins acting synergistically [9].

Only type A (CPA), C (CPA and CPB), and F (CPA and CPE) are known to affect humans, whereas all of the toxinotypes have been shown to cause disease in animals [117]. Depending on where the toxins are produced, *C. perfringens* causes histotoxic, enteric, or systemic diseases. Infection of wounds can lead to clostridial myonecrosis (gas gangrene), whereas the production of toxins in the intestines can derive into enteritis or enterocolitis. Intestinal infections may progress into enterotoxemia when toxins are absorbed into the bloodstream, affecting distant organs, such as the kidneys, lungs, or the brain [117]. A brief description of the toxins and their associated diseases are described below, however, for a more exhaustive review, please refer to previous work [118].

### 4.1. Alpha Toxin (CPA)

Chromosomic encoded CPA is produced by all *C. perfringens* isolates but in greater amounts by *C. perfringens* type A [119], which causes traumatic clostridial myonecrosis in both humans and animals. Crush-type injuries, open fractures, and penetrating wounds are common portals of entry for *C. perfringens* [3]. The disease can progress rapidly, necrotizing surrounding tissue in hours and producing a foul smell and gas bubbles characteristic of the disease (gas gangrene). Mortality rates in human adults are high, around 67% to 100% during the first 24 h, and treatment requires adequate antibiotics and surgical debridement or amputation [120]. Two toxins are implicated in this condition, CPA and PFO. While the zinc metallophospholipase CPA is essential for pathogenesis, PFO, a cholesterol-dependent cytolysin, is not required for lethality but contributes to the pathogenesis of the disease [121].

The intestinal role of CPA is controversial. It has been associated with enterotoxemia in lambs (yellow lamb disease), and enteritis or enterotoxemia in cattle, pigs, horses, and goats, however, they have been poorly documented, showing no clear evidence that CPA causes intestinal disease [118,122]. Similarly, it was believed that CPA was the main toxin responsible for causing necrotic enteritis (NE) in chickens [123]. However, it was discovered that CPA is not essential and the disease is caused by NetB [124].

### 4.2. Beta Toxin (CPB)

CPB is a plasmid-encoded, pore-forming toxin that causes severe damage to the intestinal epithelium of animals and humans. It is a trypsin-sensitive toxin produced by toxinotypes C and B. While it is widely accepted that CPB plays a crucial role in the pathogenesis of *C*. *perfringens* type C, type B pathogenesis is poorly understood, and evidence indicates that lethality arises from the combinatory effects of CPB and ETX [125].

Humans are susceptible to type C infections resulting in enteritis necroticans (EN; also known as pigbel or darmbrand). It has been associated with diets rich in trypsin inhibitors, such as large consumption of sweet potatoes, and poor food hygiene [126]. The presence of trypsin inhibitors in the intestines allows the persistence of CPB, resulting in the damage of the gut epithelium and posterior entry into the bloodstream [127]. It is a rare but fatal disease marked by abdominal pain, bloody diarrhea and necrosis of the small intestine, and toxemia and shock in acute cases [128,129]. Historical outbreaks have been reported in post-World War II Germany and non-developed countries of Southeast Asia and Africa. In Papua New Guinea, EN was the second leading cause of death in children before the introduction of immunization programs with the CPB toxoid vaccine [130]. EN has also been reported in developed countries. People with reduced trypsin production and pancreatic disease (i.e., diabetes) are at higher risk of contracting the disease. However, very few sporadic cases have been reported since 1984 in the United States, England, and Australia [131].

Animal type C infections also cause necrotic enteritis and enterotoxemia in all livestock and domestic animals, especially newborns [132]. The lack of competing flora and low levels of intestinal trypsin due to the consumption of colostrum, which has trypsin inhibitory activity, are risk factors. Type C outbreaks account for serious economic losses for the agricultural sector with fatality rates exceeding 50% and reaching 100% in unvaccinated herds [127].

*C. perfringens* type B mainly affects lambs, causing lamb dysentery, and seldom calves and foals. The production of the two toxins (CPB and ETX) in the intestine of infected animals causes extensive damage, ranging from severe necrohemorrhagic enteritis (attributed to CPB) to neurological alterations (attributed to ETX), and in peracute cases, sudden death without previous symptoms [12,125].

### 4.3. Epsilon Toxin (ETX)

Plasmid-borne ETX belongs to the aerolysin-like pore-forming toxin family and is produced by toxinotypes D and B (also CPB positive). After botulinum and tetanus toxins, ETX is the third most potent clostridial toxin and causes enterotoxemia (pulpy kidney) in domestic ruminants, especially sheep and goats, but rarely in cattle [133]. Contrary to CPB, ETX is secreted as a prototoxin and is fully activated by the action of proteases in the gastrointestinal tract of animals and by lambda toxin (a metalloprotease synthetized by *C. perfringens*) [134]. Although there are no reports of naturally occurring ETX intoxication in humans, the CDC considers the toxin as a potential bioterrorism agent [135]. *C. perfringens* type D infections are more common than type B and have been associated with sudden change in the diet of animals. The abrupt change and large consumption of feed rich in fermentable carbohydrates disrupts the gut microflora, allowing the rapid growth of *C. perfringens* [12]. Coccidiosis and parasite infections, such as tape worms, can also make the animal more susceptible to type D infections [136]. The accumulation of ETX in the intestines increases permeability of the intestinal mucosa, allowing the toxin to be absorbed into the bloodstream. It then spreads to other organs, such as brain, lungs, and kidneys, where it causes edema and necrotic lesions [137].

### 4.4. Iota Toxin (ITX)

*C. perfringens* toxinotype E carries the plasmid-encoded binary toxin ITX, which belongs to the AB toxin family together with the *C. botulinum* C2 toxin, the *Clostridium spiroforme* iota-like toxin, and the anthrax toxin of *Bacillus anthracis* [138]. ITX consists of two non-covalently linked proteins and is produced as an inactive toxin. It is activated by several proteases, such as α-chymotrypsin, pepsin, proteinase K, subtilisin, thermolysin, and lambda protease from *C. perfringens* [139]. The binding component (Ib) binds to a proteinaceous receptor, such as the lipolysis-stimulated lipoprotein receptor (LSR) and CD44 glycoprotein, allowing the internalization of the ADP-ribosyltransferase component (Ia), which disrupts the cell cytoskeleton [140,141]. *C. perfringens* type E also produces bacteriocin-like factors that favor growth over other strains and exploits the changes caused by ITX to increase adherence to enterocytes [142].

Type E infections are infrequent and misreported. Type E has been associated with hemorrhagic enteritis or sudden death in ruminants, principally in calves but rarely in lambs and goats, and is also associated with enterotoxemia in rabbits [118]. However, cross-reactivity between ITX and *C. spiroforme* iota-like toxin, which causes a similar disease in rabbits, has probably given misleading diagnosis of type E enterotoxemia in the past [143]. Currently, there are no vaccines to prevent type E outbreaks.

### 4.5. C. perfringens Enterotoxin (CPE)

CPE is a pore-forming toxin produced by *C. perfringens* type F and is responsible for food poisoning, antibiotic-associated diarrhea (ADD), sporadic diarrhea (SD), and sudden infant death syndrome (SIDS) in humans [144]. CPE infection is a common food poisoning cause in the United States [145], principally attributed to improper cooling and inadequate storing of food, allowing for germination and rapid growth. Under optimal growth conditions, *C. perfringens* has a generation time of 8 to 12 min, and as such, is one of the fastest growing known bacteria [9].

Production of CPE is co-regulated with sporulation. After the ingestion of large quantities of vegetative cells, sporulation takes place in the intestinal lumen with the subsequent release of CPE. The toxin binds to claudin receptors from the cellular tight junctions, forming complexes that create pores in the cell membrane, allowing the unregulated influx of calcium causing, in turn causing oncosis, which leads to necrosis [146]. Symptoms last for short periods no longer than 24 h and are rarely fatal. CPE causes non-foodborne diseases, including antibiotic-associated diarrhea (ADD) and sporadic diarrhea (SD), which are sometimes potentiated by the production of beta2 (CPB2) toxin. While ADD is related to antimicrobial treatments, SD is considered to be non-antibiotic related. Sudden infant death syndrome (SIDS), on the other hand, is a fatal disease seen in neonates that occurs when CPE is absorbed into the systemic circulation [144]. The role of CPE in animal disease is still not clear, although some reports suggest that it is associated with gastrointestinal disease in domestic and wild animals. There are no diagnostic criteria to establish a CPE-mediated animal disease [118].

### 4.6. Necrotic Enteritis Beta-Like Toxin (NetB)

*C. perfringens* type G produces NetB, a recently identified pore-forming toxin associated with avian necrotic enteritis (NE) [124]. Although the specific receptor for NetB has not been identified yet, NetB forms a pore allowing cations to enter the cell, causing cell rounding and lysis [147,148]. Globally, avian NE causes USD 2–6 billion of losses per year [149]. The immune state, nutrition, and intestinal health of the animals, and parasite infections such as coccidiosis, are predisposing factors for avian NE [150,151]. The disease can develop in peracute forms with deaths occurring within hours, or in clinical form, which causes diarrhea, ruffled feathers, anorexia, and depression [152,153]. The ban on the use of antibiotics has increased to subclinical necrotic enteritis (SNE) cases [154]. Although SNE mortality is very low, feed conversion is negatively affected, resulting in longer periods of growth and poor performance [154]. New preventive strategies to overcome this problem have focused on the design and development of probiotics and vaccines [155,156].

### 4.7. C. perfringens Vaccines

Similar to other pathogenic Clostridia, *C. perfringens* fermentation for vaccine production is poorly characterized and consists of growing the bacteria within a pH range of 5.5–8.0 in complex media containing animal-derived products, glucose, or other fermentable carbohydrates [157]. Purified toxin(s) or the whole culture are then chemically inactivated using formaldehyde, a time-consuming process that can lead to reduced immunogenicity [158,159]. Moreover, the ill-defined composition of the media leads to batch-to-batch variability and the continuous selection for strains that exhibit satisfactory toxin production [158].

Humans are not routinely immunized against *C. perfringens.* A human CPA toxoid vaccine was investigated in the 1930s, but inconsistent batches and antigenicity stopped its further development [160]. Nevertheless, pigbel-related deaths in Papua New Guinea were considerably diminished after the introduction of a highly effective toxoid vaccine against *C. perfringens* type C disease. The experimental human CPB toxoid vaccine was prepared by obtaining the toxoid from the formalin-inactivated cultures using ammonium sulfate precipitation [130]. Although pigbel cases are still reported in the highlands of Papua New Guinea and other areas of the country, vaccine production was stopped in 1998 [161].

Vaccination is common in the agricultural sector to avoid the debilitating diseases and sudden deaths caused by *C. perfringens* infections. Animal types B, C, and D outbreaks can be prevented by immunization with crude toxoid(s) or bacterin-toxoid(s) vaccines, which have been proven to be effective in piglets, cattle, lambs, sheep, and goats [119,160,162,163,164]. However, in some animal species, more than one round of vaccination might be required to achieve sustained immunity [165]. Commercial veterinary vaccines are polyvalent formulations of CPA, CPB, and ETX toxoid(s) or bacterin-toxoid(s) [158]. There are no commercial toxoids vaccines currently available that confer protection against ITX, CPE, or NetB. In 2005, a CPA toxoid vaccine was conditionally licensed by the FDA to control hemorrhagic bowel syndrome (HBS; or “bloody gut”) in cattle caused by *C. perfringens* type A [166]. A CPA toxoid vaccine is also commercially available to control NE in chickens. However, it has been shown that the immunization with CPA toxoid vaccines resulted in variable levels of protection [167].

The nutritional requirements of *C. perfringens* have been explored in the past [168,169]. The development of chemically defined media allowed the study of amino acids and vitamins required for *C. perfringens* proliferation. These media supported growth but no toxin production could be detected if no peptides, either synthetic or animal derived, were added [170]. Fernandez-Miyakawa et al. [171] showed that CPA production was higher in brain-heart infusion (BHI), followed by cooked meat medium (CMM), and significantly less in tryptone glucose yeast (TGY) medium. On the other hand, Murata et al. [172] reported the production of high potency CPA from *C. perfringens* PB6K in a defined medium by adding large amounts of arginine. By replacing the meat component for tryptone-salts, Chou attained CPA production in continuous cultures [173].

Besides the need for a peptides source, toxin production by *C. perfringens* type C depends on pH control, the strain used, and the presence of fermentable carbohydrates, such as dextrin, glucose, or fructose, which have been demonstrated to increase toxin production and growth [174,175]. Similarly, batch fermentations of *C. perfringens* type B are also reliant on glucose and peptone concentrations. High toxin titers have been achieved when growing *C. perfringens* type B in 5.0 g/L of meat or casein peptones in combination with 111.1 mM of glucose under controlled pH (6.5) conditions [157]. The importance of glucose or dextrin in combination with pH control has also been demonstrated in three different strains of *C. perfringens* type D [176].

At the molecular level, studies have shown that the two-component system (TCS) key virulence regulator VirS/VirR and the accessory gene regulator (Agr) quorum-sensing system are implicated in toxin regulation in *C. perfringens* [177]. The identification of these regulatory systems has helped to understand toxin production regulation in *C perfringens*. However, the extracellular signals that activate these pathways have not been elucidated. Both systems regulate CPA, PFO, CPB, and NetB production [178,179,180,181], while only the Agr system regulates ETX and CPE synthesis [179,182]. Although the molecular mechanisms that regulate ITX remain unknown, data suggest that the VirS/VirR system regulates the activity but not the expression of ITX [177].

Experimental recombinant CPA, CPB, ETX, and NetB have been studied, as well as multivalent versions combining two or more of these toxins. Formaldehyde-inactivated recombinant ETX (rETX) expressed in *E. coli* resulted in higher production of antibodies in rabbits when compared to commercial vaccines [183], while the administration of recombinant fused ETX–CPB vaccine in mice has shown an acceptable level of protection [184]. Also, a bivalent candidate vaccine comprising the C-terminal region of CPE and a subunit of the Shiga toxin (*Escherichia coli*) has shown protective and sustained immunity in mice [185]. Monovalent recombinant vaccines against CPA have also been shown to offer protective responses in mice [186,187], however, only partial protection in broiler chickens and cows were observed [159,188]. This might be associated with *C. perfringens* pathogenesis, which usually involves more than one toxin. Recombinant chimeric multivalent vaccines or a mixture of recombinant toxoids have shown an increased protective response in several animals in comparison with traditional monovalent or bivalent vaccines [158,159,189]. Similarly, recombinant NetB (rNetB) vaccine had proven to be effective when birds were challenged with mild oral dose of virulent bacteria but not against heavy in-feed challenges [190]. Moreover, significant protection was obtained when rNetB was used in combination with cell-free toxoids or bacterin preparations, indicating that avian NE, as well as other *C. perfringens* diseases, are multifactorial and the combination of antigens is necessary to evoke full immunity [190].

Altogether, development, production, and studies on the potency of recombinant vaccines have intensified in recent years. We encourage the reader to consider the review by Ferreira et al. for a more detailed insight into the advances on recombinant vaccines against *C. perfringens* [191].

## 5. Clostridium chauvoei

*C. chauvoei* causes blackleg, a fulminant necro-hemorrhagic myositis disease of cattle, sheep, and other small ruminants [192,193]. Young cattle between 6- and 24-months of age are the most susceptible to blackleg, which commonly originates as a non-traumatic endogenous infection. In sheep, the disease can occur at all ages, and it has also been associated with the infection of skin lesions caused by shearing, castration, or docking [13]. *C. chauvoei* can be found in the environment in the form of spores, which persist in soils, pastures, manure, and perished animals [194]. When the animals ingest the spores, these are then transported to muscle tissue where they reside dormant until favorable anaerobic conditions facilitate their germination, such as muscle damage caused by blunt traumas or intramuscular injections [192]. *C. chauvoei* pathogenesis relies on the release of potent toxins that cause endogenous myonecrosis and edemic lesions, giving to the affected muscles the characteristic dark color from where blackleg takes its name [192,194]. The disease progresses rapidly, causing the death of the animal within 48 h of clinical manifestation [192,195]. On the other hand, *C. chauvoei* is not considered zoonotic, and only two fatal human cases have been reported [194].

*C. chauvoei* possesses a small genome of 2.8 Mb, which shares 74% homology *with Clostridium septicum* [196]. Apart from minor variations in intergenic regions and flagellar genes, the metabolic, structural, and virulence-associated genes remain highly conserved among geographically different strains of *C. chauvoei*. This genomic stability suggests that it is a well-adapted and specialized pathogen that has reached a dead-end from an evolutional point of view [194]. However, from a molecular perspective, it is still not known how *C. chauvoei* orchestrates the spreading from the digestive tract to the skeletal muscles and pathogenesis. Several toxins and virulence factors have been described to contribute to cytolysis and hemolysis, causing the characteristic lesions of blackleg [192]. Among these, *Clostridium chauvoei* toxin A (CctA), a well-conserved toxin found in all the strains known across different continents, was identified as the major virulence factor [193]. CctA belongs to the β-barrel pore-forming toxin of the leucocidin superfamily of bacterial toxins and is secreted extracellularly causing cell lysis by perforating the cell membrane and disrupting its permeability [192,197]. Other proteins, such as sialidase, beta toxin-DNAse, hemolysin, hyaluronidase, and flagella, are also important virulence factors associated with the spread and pathogenesis of *C. chauvoei* [192,198,199].

### C. chauvoei Vaccine Production

Because blackleg advances rapidly, antibiotics are not generally used to treat the disease and vaccination is the principal prophylactic measure. Vaccination has been a common practice since 1930, especially in the bovine sector [195]. Nevertheless, although vaccination claims to be 100% effective against blackleg, the scientific evidence showing its efficacy in protecting cattle against *C. chauvoei* challenges is scant [200].

Unlike toxoid vaccines, *C. chauvoei* bacterin vaccines consist of whole formalin-inactivated bacterial cultures, which are expected to have all the components believed to be involved in blackleg pathogenesis. Commercially available *C. chauvoei* vaccines are offered as monovalent or in combination with other clostridial toxoids or bacterins. The production of the vaccine is a straightforward process consisting of growing *C. chauvoei* in fermenters for 24 h at 37 °C. Cultures are then formalized for approximately six days at 37 °C to inactivate the cells [201]. The complex medium contains casein hydrolysate, peptone, tryptone, yeast extract, and meat extract, plus L-cysteine hydrochloride, glucose, and salts [201]. Glucose excess has negative impacts on the antigenicity capacity of *C. chauvoei* cells, suggesting that carbon limitation is favorable for obtaining potent immunogenicity cultures [201].

The design of monovalent vaccines against blackleg seems to face several challenges because of the lack of knowledge about *C. chauvoei* pathogenicity and which are the specific antigens responsible for the disease. For a long time, it was believed that flagella were the main virulence factors associated with blackleg and studies using monoclonal antibodies of *C. chauvoei* flagellar proteins elicited protective immunity in mice [202,203]. However, the use of cell-free supernatant containing other extracellular antigens apart from flagella also conferred protection in a mouse model [204]. Additionally, animals vaccinated with recombinant CctA developed high immunity against *C. chauvoei* challenges, making this toxin a valuable candidate for the design of a toxoid vaccine against blackleg [193].

## 6. Clostridium septicum

*C. septicum* is the major etiological agent of non-traumatic or spontaneous myonecrosis in humans [205] and braxy in sheep and calves [13,206]. It is also responsible for causing malignant edema (gas gangrene) in ruminants [207] and clostridial or gangrenous dermatitis in poultry [208,209]. However, other *Clostridium* species, such as *C. sordellii*, *C. novyi* type A, and *C. perfringens* type A, are also known to cause the latter animal diseases [207,208,210].

In humans, spontaneous myonecrosis has been associated with damaged intestinal walls and several other factors, such as occult or known malignancy, leukemia, clinical neutropenia, drug immunosuppression, diabetes, radiation therapy, and chemotherapy, which might generate the conditions that allow bacteria proliferation [211,212,213,214]. The disease has higher mortality rates than other clostridial infections [215,216], with death occurring in the first 24 h [217]. *C. septicum* lethality might be related to its higher virulence and aerotolerance compared to other *Clostridium* species that cause human myonecrosis [211]. As there are no prophylactic measures available to humans, treatment is based on antibiotic therapy [205] and surgical debridement [217].

*C. septicum* infections are responsible for severe economic losses to the livestock and poultry industries [208,218]. Additionally, the current halt to the use of antibiotics as growth promoters could potentially increase the cases of clostridial dermatitis in poultry [209]. Malignant edema in livestock have been associated with the infection of wounds by *C. septicum* due to poor hygiene practices including vaccination, castration and dehorning as well as other medical and surgical procedures [13,219,220]. On the other hand, the ingestion of frozen pastures has been related to cases of braxy [12]. The portals of entry for *C. septicum* infection in poultry are still poorly characterized [221]. However, it is believed that infections can arise endogenously from gastric damage and exogenously through skin breaks [208].

*C. septicum* is known to produce several toxins [222], but its pathogenesis is principally mediated by the lethal alpha toxin (ATX) [223]. ATX is encoded by the *csa* gene and secreted as a prototoxin of 46 kDa, which undergoes proteolytic cleavage to form an active toxin of 43 kDa [224]. The pore-forming ATX causes cell oncosis [225] and it is structurally related to the aerolysin of *Aeromonas hydrophila* [226] and ETX of *C. perfringens* type B and D [224].

*C. septicum* is phylogenetically related to *C. chauvoei* [196] and harbors a homologous *agrD* gene, which is known to regulate virulence in other pathogenic *Clostridium* [7]. However, molecular studies focusing on the regulation of ATX production are non-existent.

### C. septicum Vaccine Production

Vaccination with bacterin-toxoid or purified toxoid preparations has proven to be an effective protective measure against malignant edema, clostridial dermatitis, and braxy [227,228,229]. Vaccines are produced by anaerobically growing *C. septicum* in a complex medium, such as meat peptones, and in literature in brain heart infusion, containing 0.3% glucose and 0.05% L-cysteine hydrochloride at 37 °C and pH between 7.2 and 7.4 for 18 to 24 h [227,228,230]. For the toxoid vaccine, ATX is harvested at the lowest point in redox potential, concentrated, purified, and inactivated in formalin 0.4% at 37 °C for 4 days [227,231]. Nutritional studies in *C. septicum* have shown that serine, lysine, nicotinamide, and thiamine are required for growth [232]. Nonetheless, at the large scale, ATX production is hampered by low biomass and low toxin titers [233,234]. Attempts to increase toxin yields have been successful in dialyzed cultures [233] and in glucose-limited batch fermentations with controlled pH [234], suggesting that carbon limitation favors toxin production.

Finally, a recombinant, noncytolytic ATX has been shown to elicit protection in turkeys. Its manufacture is inexpensive compared to traditional clostridial toxoid vaccines [235], making it a good candidate for further recombinant vaccine development.

## 7. Clostridium novyi

*Clostridium novyi*, previously known as *C. edematiens*, causes disease in humans and animals and can be classified into three different types, namely A, B, and C, based on toxin production (Table 1). *C. novyi* type A causes myonecrosis (gas gangrene, malignant edema) in humans and animals [13], while type B principally causes infectious necrotic hepatitis (black disease) in ruminants, pigs, and horses [236,237,238,239]. Type B human infections are infrequent [240,241]. Conversely, type C is non-pathogenic [242].

Human myonecrosis caused by *C. novyi* type A infections are uncommon and have been reported in injection drug users [243,244,245]. Open wounds are the common portal of entry for *C. novyi* infections [246]. For instance, repeated injections with contaminated heroin lead to local-tissue ischemia and necrosis, creating a favorable environment for *C. novyi* spores to proliferate [244]. Other factors, such as immunosuppression, cancer, liver abscess, chemotherapy, or poorly controlled diabetes, might contribute to *C. novyi* propagation [240,241,247]. The mortality of *C. novyi* infections is high (~50%) [246] and treatment consists of intensive antibiotic therapy and aggressive surgical debridement [247]. At present, there are no commercially available vaccines to protect humans against *C. novyi* infections.

Animal wounds infected with *C. novyi* type A can also cause myonecrosis (gas gangrene) [210,248], which results in massive edemas due to the toxin effect on vascular permeability, which is described in more detail in the *C. perfringens* section. However, *C. novyi* type A cases are less frequent than other infection of histotoxic clostridia [249], or are under-reported. Treatment of the disease is frequently not successful [210,250]. *C. novyi* type B causes black disease or infectious necrotic hepatitis, a well-documented disease in animals [236]. The ingestion of *C. novyi* spores tends to accumulate in distant organs, such as the liver, spleen, and bone marrow [20], without showing apparent symptoms. In ruminants, *C. novyi* outbreaks are caused by the migration of immature liver flukes, which damage the liver, creating suitable anaerobic conditions for spore germination, growth and toxin production [251]. Other low-grade infectious diseases, such as pneumonia or metritis in swine, can also trigger *C. novyi* spore germination and subsequent disease [237]. The rapid necrosis and toxemia lead to sudden death or death between 12–48 h, for which a successful therapy has not been described [20,237]. The darkening of the subcutaneous tissues and blackening of the carcasses after death are the hallmarks from which black disease takes its name [251].

*C. novyi* produces many different toxins [13]. However, pathogenesis of both *C. novyi* type A and B is principally mediated by the lethal and necrotizing alpha toxin (TcnA), which belongs to the family of large clostridial cytotoxins [252], along with toxin A and B of *C. difficile* and the lethal toxin of *C. sordellii* [253]. TcnA glycosyltransferase activity irreversibly modifies small GTP-binding proteins affecting the cell actin cytoskeleton, causing cell rounding and retraction [254,255]. Also, type B produces beta toxin, a phospholipase serologically identical to the beta toxin of *C. hemolyticum* but different from the phospholipases of *C. novyi* type A, *C. perfringens* type A, and *C. bifermentans* [256]. This toxin is further explained in the *C. hemolyticum* section below.

Molecular studies on toxin production regulation in *C. novyi* are scarce. TcnA is encoded within a prophage genome in both *C. novyi* toxinotypes [257]. This bacteriophage is believed to regulate toxin production and morphology [258,259,260]. Moreover, homologous genes or a novel *C. perfringens* toxin regulator (CPE1446-CPE1447) [261] and an AgrD quorum sensing peptide (Cno-19402) [7] are found in *C. novyi.*

### C. novyi Vaccine Production

The economic losses caused by *C. novyi* infections can be prevented by vaccination. *C. novyi* toxoid vaccines are proven to be effective in animals [227,262,263,264] and humans [263]. However, human vaccines are not produced any more. Research on *C. novyi* culture optimization for toxin production dates back to the 1930s, with limited publication on this topic reported recently.

Early studies showed that growth of *C. novyi* required strict anaerobiosis in a medium containing amino acids, with components of the vitamin B complex and peptides coming from meat and other peptones [265,266]. The presence of sugars in the fermentation medium was firstly reported to negatively affect toxin titers [265]. However, it was later shown that the addition of sugars was indispensable for toxin production [267]. *C. novyi* type A can ferment glucose, dextrin, and maltose, while type B strains can ferment glucose, inositol, maltose, and mannose [268]. The use of 5% chopped meat particles, 3% peptone, 0.5% phosphate, and 1% maltose in meat infusion broth showed higher toxin production than previously reported media [267]. Likewise, alternative fermentation methods, such as dialysis flasks containing brain heart infusion with 0.75% Tween 80 and 2% glycerol, also produced high toxin titers [269]. *C. novyi* is grown for 18 to 72 h at 37 °C and inactivation of TcnA is achieved by incubation with 0.4% formalin at 37 °C for three days [227]. A more recent study showed that modeling the difficult *C. novyi* fermentation process using computational tools allowed optimization of toxin productivity, cell density, and fermentation time [260].

## 8. Clostridium hemolyticum

Bacillary hemoglobinuria (BH), or red water, is a disease caused *by Clostridium hemolyticum*, previously known as *C. novyi* type D. The disease affects cattle, and has occasionally been reported in sheep and goats [270,271,272,273]. Human infections are extremely rare [274,275]. BH is common during dry seasons and in cattle that graze in pastures infested with liver flukes [276]. Similar to *C. novyi*, the ingested spores of *C. hemolyticum* are absorbed by the intestine and transported to the liver where they reside without causing disease. The migration of immature liver flukes causes local damage and anaerobiosis, which prompts *C. hemolyticum* germination and pathogenesis [270]. BH is characterized by severe hemolytic anemia with clinical symptoms (hemoglobinuria, jaundice, and fever) that frequently last for 10 to 12 h and rarely more than 3–4 days [276,277]. The sudden death of animals without clinical signs is also common [272]. The lethality of *C. hemolyticum* infections can reach 80% to 100% [276]. Some reports have shown recovery by using antibiotic therapy [278,279]; however, due to the rapid evolution of the disease, fatal cases are more common.

The pathogenesis of *C. hemolyticum* is mediated by the beta toxin, a lethal hemolytic and necrotizing phospholipase type C serologically identical to the beta toxin of *C. novyi* type B [13,256]. However, *C. hemolyticum* produces more beta toxin than *C. novyi* type B [280,281]. The toxin provokes hepatocytes and erythrocytes destruction by hydrolysis of phosphatidylcholine into phosphocholine and diacylglyceride, as well as causing platelet aggregation and vascular permeability [270,277].

### C. hemolyticum Vaccine Production

BH can be prevented by vaccination with the *C. hemolyticum* bacterin-toxoid [276,277]. Studies on different media showed that high toxin titers were obtained in media consisting of 5% trypticase peptone, 0.5% proteose peptone, 0.5% yeast extract, 0.5% glucose, 0.05% cysteine hydrochloride, and 0.1% sodium thioglycollate [282]. Higher toxin titers were obtained after 18 h of growth at 37 °C in dialysis cultures than in batch fermentations [282]. However, current bacterins do not induce a measurable amount of antitoxin titers, and frequent vaccination is required to assure protection against the disease [277]. It has been shown that the inactivation of the beta toxin with formaldehyde destroys the immunogenicity of the toxin, but the addition of glycine seems to stabilize the denaturation process, resulting in immunogenic toxoids [276]. On the other hand, vaccination with crude formalinized recombinant beta toxin has shown a protective effect in guinea pigs [277].

## 9. Concluding Remarks

Clostridial toxoid or bacterin-toxoid vaccines are of high importance as prevention of animal clostridiosis fully relies on immunization. However, the large-scale production of clostridial toxoids and bacterins have remained unchanged since the mid-1900s, with the industry still facing batch-to-batch variability. The necessity to use complex media containing animal or soy-derived products, due to the link between peptide presence and toxin production, have hampered the study and understanding of *Clostridium* toxinogenesis. There have been no reports of a chemically defined medium that gives high and consistent toxin titers in *Clostridium*. Furthermore, the knowledge of toxin regulation in several of these pathogens is also very limited, especially when compared to the human pathogen *C. difficile.* Genetic engineering studies would help to elucidate the molecular mechanisms that regulate toxin production. The lack of genetic studies in pathogenic *Clostridium* is, understandably, attributed to regulatory constraints. Due to the spore-forming nature of *Clostridium* and the potency of the toxins, strict regulations should be applied to prevent the spread of highly toxic engineered mutants. The insertion of a reporter gene instead of the toxin gene is a safe way of doing this. The lack of recent literature on this topic is also linked to the efficacy and the high sales value of these vaccines, forcing manufacturers to maintain new developments and improvements as trade secrets.

The new generation of vaccines is proving to be an effective alternative to conventional vaccine manufacturing. Although beyond the scope of this review, glycoconjugate vaccines are among the safest and most successful vaccines in the last 40 years [283]. The development of glycoconjugate vaccines consisting of a polysaccharide (e.g., cell-surface protein) covalently linked to a carrier protein, studied in detail in *C. difficile*, represent potential candidate vaccines to fight *C. difficile* invasion and colonization [284,285]. However, not all pathogenic *Clostridium* species are invasive, therefore further studies should be conducted to understand host-pathogen interactions in other *Clostridium* species. Other vaccination strategies, such as mucosal immunization, offer many advantages over traditional vaccination [286]. Intranasal vaccines consisting of BoNT/A toxoid with an adjuvant [287], BoNT-Hc/A, or tetanus toxoid administered using cCHP nanogel [288], have proven to elicit protective immunity in mice. On the other hand, recombinant vaccines offer greater purity of the final product and some recombinant toxins (e.g., subunits) do not need inactivation steps, shortening the production process and preventing residual formaldehyde in the final vaccine preparation.

Furthermore, it is possible to produce fusion-toxins by using immunogenic epitopes from different toxins in a single expression system. However, recombinant clostridial vaccines are not commercially available yet, and their production still presents several disadvantages to traditional toxoid vaccines. Importantly, given the immunity that the first generation of vaccines have provided to the human and animal population worldwide, it is unlikely that anything will change for the established vaccines and new developments are only likely to be applied to the new generation of vaccines. For all new developments, it is important to consider purification steps, antigen presentation and concentration, protein insolubility and refolding, and the generation of unspecific immune response to portions of the recombinant proteins that are not relevant to fight the disease.

## Figures and Tables

**Table 1:** Classification of pathogenic clostridia by toxin(s) produced, the disease they cause, current protective method(s), and recombinant vaccine research.

Pathogenic Strain	Type	Toxin	Disease	Commercial Toxoid or Bacterin-Toxoid Vaccine	Recombinant Vaccine Research
*C. botulinum*	Group I	BoNT serotypes A, B, E, and F	Botulism (human and animal)	Pentavalent BoNT/A–E toxoids (for people at high risk or exposed to the toxin). Discontinued. BoNT/B toxoid (horses)	rBV A/B Withdrawn from human clinical trial. rHC BoNT/A/B/E
	Group II	BoNT serotypes B, E, and F	Botulism (human and animal)	-	-
	Group III	BoNT serotypes C and D	Botulism (human and animal)	Bivalent BoNT/C-D toxoids (livestock)	rHC BoNT/C
*C. tetani*	-	TeNT	Tetanus (human and animal)	TeNT toxoid (human and animal)	TeNT-HC
*C. perfringens*	Type A	CPA	CPA: Myonecrosis (human and animal).	Monovalent CPA toxoid vaccine (cattle and poultry).	rCPA
	Type B	CPA, CPB, ETX	CPB: Necrohemorrhagic enteritis (animal) ETX: Dysentery (lambs) and enterotoxemia or pulpy kidney (domestic ruminants)	CPB and ETX toxoid(s) or bacterin-toxoid(s) vaccine (animal)	rCPB rETX r- fused ETX-CPB vaccine
	Type C	CPA, CPB,	CPB: Enteritis necroticans or pigbel (human) and necrotic enteritis, enterotoxemia (animal)	Experimental CPB toxoid vaccine (for people in Papua New Guinea). Discontinued. CPB toxoid or bacterin-toxoid (animals)	rCPB
	Type D	CPA, ETX,	ETX: Enterotoxemia or pulpy kidney (domestic ruminants)	ETX toxoid or bacterin-toxoid (animal)	rETX
	Type E	CPA, ITX,	ITX: Hemorrhagic enteritis (ruminants) and enterotoxemia (rabbits). Not confirmed.	-	-
	Type F	CPA, CPE	CPE: food poisoning, antibiotic-associated diarrhea, sporadic diarrhea, and sudden infant death syndrome (SIDS) (humans), and gastrointestinal disease (animals)	-	rC-terminal CPE r-fused C-terminal CPE with Shiga toxin B subunit
	Type G	CPA, NetB	NetB: Necrotic enteritis (poultry)	-	rNetB
*C. chauvoei*	-	CctA	Blackleg (cattle, sheep, and other small ruminants)	Bacterin-toxoid vaccine (animal)	rCctA
*C. septicum*	-	ATX	Spontaneous myonecrosis (human) Braxy (sheep and calves) Malignant edema (ruminants) Gangrenous dermatitis (poultry)	Toxoid or bacterin-toxoid vaccine (animal)	rATX
*C. novyi*	A	TcnA	Myonecrosis (human and animal)	-	-
	B	Beta toxin	Black disease (ruminants, pigs, and horses)	Toxoid vaccine (animal)	-
*C. hemolyticum*	-	Beta toxin	Bacillary hemoglobinuria (cattle and occasionally in sheep and goats)	Bacterin-toxoid vaccine (animal)	r-beta toxin

BoNT: botulinum neurotoxin. TeNT: tetanus neurotoxin. CPA: *C. perfringens* Alpha toxin. CPB: *C. perfringens* Beta toxin. ETX: *C. perfringens* Epsilon toxin. ITX: *C. perfringens* Iota toxin. NetB: Necrotic enteritis Beta-like toxin. CPE: *C. perfringens* Enterotoxin. CctA: *C. chauvoei* toxin A. ATX: *C. septicum* lethal Alpha toxin. TcnA: *C. novyi* Alpha toxin. r-: recombinant. HC: Heavy chain. rBV: recombinant botulinum vaccine. rHC: recombinant heavy chain.
